# An Optimization Path for Sb_2_(S,Se)_3_ Solar Cells to Achieve an Efficiency Exceeding 20%

**DOI:** 10.3390/nano14171433

**Published:** 2024-09-02

**Authors:** Xiaoyong Xiong, Chao Ding, Bingfeng Jiang, Guanggen Zeng, Bing Li

**Affiliations:** 1 College of Materials Science and Engineering, Sichuan University, Chengdu 610064, China; 2 Institute of New Energy and Low-Carbon Technology, Sichuan University, Chengdu 610065, China; 3 College of Intelligent Systems Science and Engineering, Hubei Minzu University, Enshi 445000, China

**Keywords:** Sb_2_(S,Se)_3_ solar cell, internal resistance, nonradiative recombination, fill factor, open-circuit voltage

## Abstract

Antimony selenosulfide, denoted as Sb_2_(S,Se)_3_, has garnered attention as an eco-friendly semiconductor candidate for thin-film photovoltaics due to its light-absorbing properties. The power conversion efficiency (PCE) of Sb_2_(S,Se)_3_ solar cells has recently increased to 10.75%, but significant challenges persist, particularly in the areas of open-circuit voltage (*V*_oc_) losses and fill factor (FF) losses. This study delves into the theoretical relationship between *V*_oc_ and FF, revealing that, under conditions of low *V*_oc_ and FF, internal resistance has a more pronounced effect on FF compared to non-radiative recombination. To address *V*_oc_ and FF losses effectively, a phased optimization strategy was devised and implemented, paving the way for Sb_2_(S,Se)_3_ solar cells with PCEs exceeding 20%. By optimizing internal resistance, the FF loss was reduced from 10.79% to 2.80%, increasing the PCE to 12.57%. Subsequently, modifying the band level at the interface resulted in an 18.75% increase in *V*_oc_, pushing the PCE above 15%. Furthermore, minimizing interface recombination reduced *V*_oc_ loss to 0.45 V and FF loss to 0.96%, enabling the PCE to surpass 20%. Finally, by augmenting the absorber layer thickness to 600 nm, we fully utilized the light absorption potential of Sb_2_(S,Se)_3_, achieving an unprecedented PCE of 26.77%. This study pinpoints the key factors affecting *V*_oc_ and FF losses in Sb_2_(S,Se)_3_ solar cells and outlines an optimization pathway that markedly improves device efficiency, providing a valuable reference for further development of high-performance photovoltaic applications.

## 1. Introduction

With the increasing demand for renewable energy and a heightened focus on environmental sustainability, photovoltaic (PV) technology has emerged as a crucial method for converting solar energy into electricity. Thin-film solar cells, which utilize inorganic materials such as Cu(In,Ga)Se_2_ (CIGS) and CdTe, have been commercialized, offering excellent power conversion efficiency (PCE) and especially lighter weights compared to silicon-based solar cells. However, the use of expensive rare earth elements (Ga and Te) and the toxicity of Cd present challenges to the broader application of these thin-film solar cells. Recently, researchers have shown significant interest in antimony selenosulfide semiconductor materials, Sb_2_(S_,_Se)_3_, due to their nontoxic nature, profuse existence in the Earth’s crust, excellent ambient stability, large dielectric constant (*ε_r_* > 15), elevated relative absorption coefficient (*α* > 10^5^ cm^−1^), and adjustable bandgap (*E*_g_) ranging from 1.1 to 1.7 eV [1,2]. Despite substantial efforts by researchers to enhance the PCE of Sb_2_(S,Se)_3_ solar cells, the current champion PCE for Sb_2_(S,Se)_3_ thin-film solar cells remains at 10.75% [3,4]. Nevertheless, these devices still suffer from a substantial open-circuit voltage (*V*_oc_) loss (defined as *E*_g_/*q* − *V*_oc_) of approximately 0.8 V, and a fill factor (FF) loss (defined as the difference between the FF of ideal solar cells and the FF observed in practice) of approximately 15%. Moreover, there is still a considerable gap to bridge before reaching the Shockley–Queisser (S–Q) limit, which is based on the detailed balance principle and predicts a maximum efficiency of 33% for single-junction solar cells under standard illumination conditions [5]. Therefore, continued research endeavors aimed at mitigating these *V*_oc_ and FF losses in Sb_2_(S,Se)_3_ solar cells are crucial to advancing towards this theoretical benchmark.

To achieve exceptional performance in Sb_2_(S,Se)_3_ solar cells, we used a solar cell capacitance simulator (SCAPS) to analyze the limiting factors, including series resistance (*R*_s_), shunt resistance (*R*_sh_), bulk defects, interface recombination, and energy level arrangements between different functional layers. SCAPS is a powerful solar cell simulator that has been validated by numerous studies for accurately adjusting the parameters of each functional layer and interface, simulating photovoltaic device performance, optimizing device structures, and obtaining high-efficiency solar cells. For example, in our previous work [6], we achieved a PCE of over 15% by utilizing SCAPS for guiding interface engineering strategies and optimizing the design of PbS colloidal quantum dot solar cells. Similarly, Chen et al. [7] successfully prepared Sb_2_Se_3_ solar cells with a certified efficiency of 6.5% using EDT-treated PbS colloidal quantum dots as hole transport layers (HTLs), guided by SCAPS simulation results. Bertens et al. [8] used SCAPS to predict the effect of doping concentration in the active layer on the performance of PbS colloidal quantum dot solar cells and achieved actual devices that matched the predicted performance.

This exhaustive investigation delves into the multifaceted factors that currently impede the enhancement of Sb_2_(S,Se)_3_ solar cell efficiency, with the ultimate goal of identifying optimization strategies to attain a PCE exceeding 20%. By utilizing numerical methods, we constructed a theoretical model for the most efficient Sb_2_(S,Se)_3_ solar cell structure reported thus far, featuring a fluorine-doped tin oxide (FTO)/CdS/Sb_2_(S,Se)_3_/Spiro-OMeTAD/Au configuration. Initially, through meticulous optimization of *R*_s_ and *R*_sh_, we significantly reduced the FF loss from 10.79% to 2.80%, increasing the PCE from 10.72% to 12.18%. For the device exhibiting 12.18% efficiency, by refining the alignment of the conduction band between the electron transport layer (ETL) and the absorber layer, we achieved a notable increase in *V*_oc_, increasing it from 0.64 V to 0.76 V. This adjustment minimized the *V*_oc_ loss and increased the PCE to 15.31%. Building upon this improved device, we further optimized the interface recombination, achieving a substantial 43% decrease in the *V*_oc_ loss relative to that of the initial device, with FF losses decreasing below 1% and a substantial increase in the PCE to 23.75%. In the final phase of optimization, we targeted the absorber defects and thickness, resulting in an increase in the PCE to 26.77%, accompanied by an increase in the short-circuit current density (*J*_sc_) to 30.93 mA·cm^−2^. The simulation results revealed that under conditions of low *V*_oc_ and FF, the internal resistance exerted a more substantial constraint than nonradiative recombination on the FF. Additionally, the spike-like energy band offset (EBO) increase at the FTO/ETL interface could impede the formation of a flat or slightly spiked EBO at the ETL/absorber interface, thus impeding further enhancements in the PCE. Importantly, mitigating interface recombination at the ETL/absorber interface has emerged as a paramount strategy for enhancing the overall performance of Sb_2_(S,Se)_3_ solar cells, surpassing the importance of reducing recombination at the absorber/HTL interface. Remarkably, as the absorber layer thickness increased to 600 nm, the Sb_2_(S,Se)_3_ solar cell achieved a noteworthy *J*_sc_ exceeding 30 mA·cm^−2^, consequently achieving a PCE of 26.77%. This study elucidates the critical factors influencing the performance of Sb_2_(S,Se)_3_ solar cells, providing valuable guidance for further experimental work in this field.

## 2. Methods and Details

### 2.1. Structure of the Solar Cells

**Figure 1:**
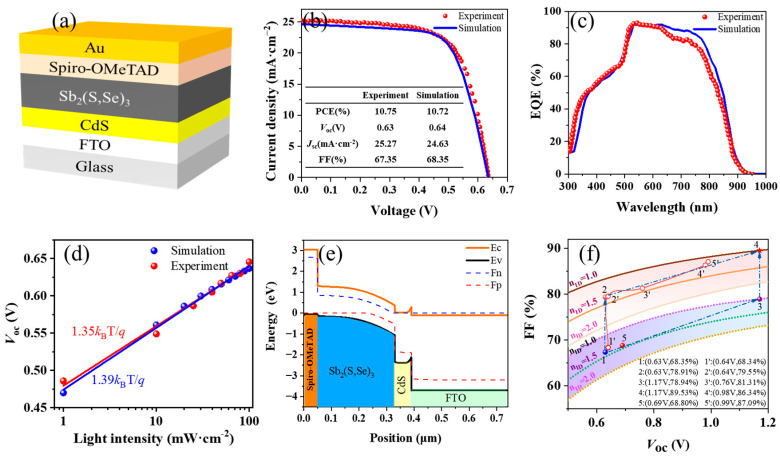
(**a**) Schematic illustrations depicting the simulated device. (**b**) *J*–*V* curves*,* (**c**) EQE spectra, and (**d**) the ideal factor (*n*_ID_) comparison between the experimental and simulated devices. (**e**) Energy band structure of the Sb_2_(S,Se)_3_ solar cell. (**f**) The FF_m_ (solid lines) and FF (dotted lines) versus *V*_oc_ for different *n*_ID_ values. Experimental data from the literature [4].

The proposed device structure, depicted in Figure 1a, follows this configuration: FTO/CdS/Sb_2_(S,Se)_3_/Spiro-OMeTAD/Au. This setup has been widely adopted in various high-efficiency Sb_2_(S,Se)_3_ solar cells [3,4,9,10,11,12,13,14,15]. FTO (SnO_2_:F) serves as a transparent conductive oxide for charge collection, CdS acts as the ETL, Sb_2_(S,Se)_3_ functions as the absorber layer, Spiro-OMeTAD is utilized as the HTL, and Au is used as the electrode for charge collection. 

### 2.2. Simulation Principles and Details

SCAPS has been meticulously crafted and generously offered by the University of Gent [16,17]. It is specifically designed to simulate a wide array of solar cells, including crystalline silicon, thin films, and emerging photovoltaic technologies. The software incorporates sophisticated numerical algorithms to solve the relevant physical equations governing charge transport, recombination, and optical properties within the device. 

The primary function of SCAPS is to solve the Poisson equation related to the electrostatic potential (*ψ*) and the continuity equation controlling the dynamics of free electrons and holes in one-dimensional heterojunctions [18]. The specific Equations are as follows:

(1) $$\frac{\partial }{\partial x}\left(\varepsilon \frac{d\psi }{dx}\right)+q[p-n+N_{D}-N_{A}+\rho_{p}-\rho_{n}]=0$$

(2) $$-\frac{1}{q}\frac{\partial J_{n}}{\partial x}-R_{n}+G=\frac{\partial n}{\partial t}$$

(3) $$-\frac{1}{q}\frac{\partial J_{p}}{\partial x}-R_{p}+G=\frac{\partial p}{\partial t}$$

(4) $$J_{n}=-\mu_{n}n\frac{dE_{Fn}}{dx},\;\;J_{p}=\mu_{p}p\frac{dE_{Fp}}{dx}$$

where *ε* represents the dielectric constant; *q* represents the electron charge; *p* and *n* symbolize the free hole and electron densities; *N*_A_ and *N*_D_ represent the densities of the acceptor and donor types, respectively; *ρ*_n_ and *ρ*_p_ indicate the densities of trapped electrons and holes, respectively; *J*_n_ and *J*_p_ denote the current densities of the electrons and holes, respectively; *G* designates the photogeneration rate; and, *R*_n_ and *R*_p_ are defined as the recombination rates of the electrons and holes, respectively. Additionally, *μ*_n_ and *μ*_p_ denote the electron and hole mobilities, respectively, and *E*_Fn_ and *E*_Fp_ represent the acceptor and donor quasi-Fermi levels, respectively.

To model the device structure via SCAPS, we compiled material parameters obtained from calculations and the relevant literature [3,4,9,18,19,20,21,22,23,24,25], which are summarized in Table S1 (see the Supplementary Material). Additionally, we standardized the thermal velocities of electrons and holes to 10^7^ cm·s^−1^ across all the layers. The specific bulk defects for the absorber layer are presented in Table S2, while Table S3 provides the detailed interface characteristics essential for the simulation. The absorption spectral data for FTO and CdS were obtained from the default file provided by SCAPS software (version 3.3.10; University of Gent, Belgium) support, and the absorption spectral data for the Sb_2_(S,Se)_3_ material obtained from reference [4] were utilized in the simulation process. All the simulations were carried out at 300 K, neglecting radiative and Auger recombination, under the AM 1.5 G spectrum.

### 2.3. Device Validation and Optimization Route

Simulation 1 of the Sb_2_(S,Se)_3_ solar cell was established utilizing the parameters outlined in Tables S1–S3 of the Supplementary Material. When *R*_s_ = 3 Ω·cm^2^ and *R*_sh_ = 375 Ω·cm^2^, the simulation results strongly agree with the experimental findings reported in [4], particularly the current density–voltage (*J*–*V*) characteristic curves and external quantum efficiency (EQE) spectra, as evident in Figure 1b,c, respectively. To further confirm the accuracy of the model, a comparative analysis was conducted on how *V*_oc_ correlates with variations in light intensity. According to the data presented in Figure 1d, the ideal factor (*n*_ID_) values of the experimental and simulated devices are 1.35 and 1.39, respectively, revealing strong consistency between the measurements and simulations. Additionally, an *n*_ID_ value between 1 and 2 indicates that interface recombination predominates in Sb_2_(S,Se)_3_ solar cells [26]. As depicted in Figure 1e, the band alignment diagram clearly illustrates that the Sb_2_(S,Se)_3_ absorber layer is fully depleted, indicating a strong electric field throughout the entire layer. This robust electric field serves as a driving force, greatly enhancing the separation efficiency of photogenerated electron–hole pairs.

In our simulated device, the Sb_2_(S,Se)_3_ absorber has an *E*_g_ of 1.43 eV, which approaches the nearly ideal bandgap (*E*_g_ = 1.34 eV) of the S–Q efficiency limit [5,27]. However, the actual device performance only reaches a maximum efficiency of 10.75%, which is significantly below the S–Q efficiency limit. For instance, at an *E*_g_ of 1.43 eV, the S–Q limit predicts a PCE of 32.54% with a *V*_oc_ of 1.17 V and an FF of 89.53% [5]. A strong correlation exists between FF and *V*_oc_, where the maximum achievable FF (FF_m_) theoretically depends solely on *V*_oc_ [3,28,29], assuming a negligibly small *R*_s_ (*R*_s_ ≈ 0) and an ideal *R*_sh_ (*R*_sh_ → ∞).

(5) $${FF}_{m}=\frac{\nu_{oc}-ln(\nu_{oc}+0.72)}{\nu_{oc}+1},\;\;with\;\nu_{oc}=\frac{qV_{oc}}{n_{ID}k_{B}T}$$

The relationship between FF_m_ and *V*_oc_ is primarily influenced by *n*_ID_, which represents the nonradiative recombination rate. However, in actual devices, *R*_s_ and *R*_sh_ cannot be neglected. *R*_s_ and *R*_sh_ are considered in Equation (5), as shown in Equation (6) [30,31].

(6) $$FF={FF}_{s}\left(1-\frac{\nu_{oc}+0.7}{\nu_{oc}}\;\frac{{FF}_{s}}{r_{sh}}\right),\;\;with\;{FF}_{s}={FF}_{m}\left(1-1.1r_{s}\right)+\frac{r_{s}^{2}}{5.4},\;\;r_{sh}=\frac{J_{sc}R_{sh}}{V_{oc}}\;and\;r_{s}=\frac{J_{sc}R_{s}}{V_{oc}}$$

Applying Equations (5) and (6), the FF and FF_m_ of Sb_2_(S,Se)_3_ solar cells were determined on the basis of empirical *V*_oc_–FF plots, where the *n*_ID_ value was varied from 1 to 2. According to Figure 1f, with *R*_s_ = 0 Ω·cm^2^ and *R*_sh_ = 10^7^ Ω·cm^2^, at a *V*_oc_ of approximately 0.63 V (that is, from point 1 to point 2 on the graph), the calculated FF increased from 68.35% to 78.91%. On the other hand, by decreasing *n*_ID_ from 1.39 to 1.0, the FF increased from 68.35% at 0.63 V to 79.94% at the S–Q limit, with a *V*_oc_ of 1.17 V (that is, moving from point 1 to point 3). On the basis of these findings, after the respective optimizations, the FF constrained by the internal resistance (78.91%) was lower than that constrained by the nonradiative recombination (79.94%). Consequently, within the regimes of low *V*_oc_ and FF, the internal resistance exerted a more significant limiting effect on the FF than nonradiative recombination did. These results highlight the rationale behind implementing a phased strategy employing staged optimization to reduce *V*_oc_ loss and FF loss in Sb_2_(S,Se)_3_ solar cells. This strategy involves initially optimizing internal resistances before addressing nonradiative recombination in Sb_2_(S,Se)_3_ solar cells (that is, from point 1 to point 2 and then to point 4, as illustrated in Figure 1f). Additionally, point 5 in Figure 1f represents the high-efficiency Sb_2_(S,Se)_3_ solar cell with the current lowest *V*_oc_ loss [3], whereas points 1′–5′ represent the results from five simulated devices obtained following our designed optimization route.

## 3. Results and Discussion

### 3.1. Optimization of the Series and Shunt Resistances

**Figure 2:**
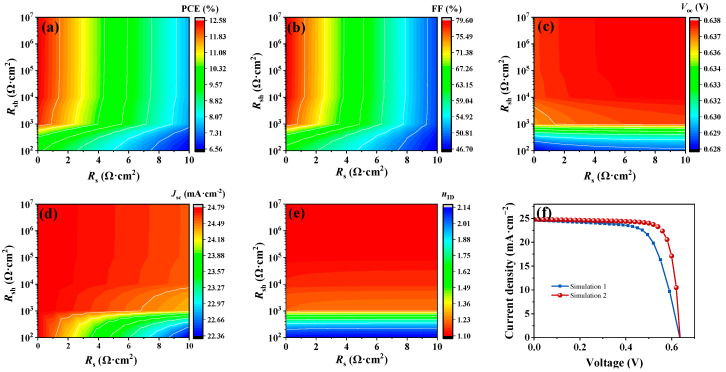
Simulated results for (**a**) PCE, (**b**) FF, (**c**) *V*_oc_, (**d**) *J*_sc_, and (**e**) *n*_ID_ with varying *R*_s_ and *R*_sh_. (**f**) The *J*–*V* curves for Simulation 1 versus Simulation 2.

The series and shunt resistances are crucial factors influencing the performance of solar cells. Simulation 1 effectively replicates the experimental *J*–*V* curves, EQE spectra, and ideality factor (*n*_ID_), aligning closely with published data [4]. This validation highlights the predictive accuracy of subsequent simulations in predicting experimental outcomes. To explore the detailed effects of *R*_s_ and *R*_sh_, *R*_s_ was adjusted to fall within the range of 0 to 10 Ω·cm^2^, and the range specified for *R*_sh_ extended from 10^2^ to 10^7^ Ω·cm^2^. The simulation calculation was carried out while the other parameters in Simulation 1 remained unchanged. As illustrated in Figure 2a–d, changes in *R*_s_ have a more pronounced effect on the PCE and FF than variations in *R*_sh_ do. When *R*_s_ decreased to 0 Ω·cm^2^ and *R*_sh_ was maintained at 10^5^ Ω·cm^2^, the FF notably increased from 68.35% to 79.55%. Conversely, the *V*_oc_ and *J*_sc_ values are least affected by changes in *R*_s_, which is consistent with previous reports [32,33]. Moreover, Figure 2e demonstrates that *n*_ID_ remains stable with variations in *R*_s_ when *R*_sh_ remains constant but shifts noticeably from 2.14 to 1.10 as *R*_sh_ varies from 10^2^ Ω·cm^2^ to 10^5^ Ω·cm^2^. Compared with Simulation 1, the *J*–*V* curve of Simulation 2 (*R*_s_ decreased to 0 Ω·cm^2^ and *R*_sh_ increased to 10^5^ Ω·cm^2^) significantly improved the FF, whereas the PCE also increased from 10.72% to 12.57% (Figure 2f). This enhancement primarily results from a marked decrease in FF loss, decreasing from 10.79% to 2.80% as detailed in Table 1, emphasizing the critical role of *R*_s_ and *R*_sh_ optimization in enhancing solar cell performance. 

The findings underscore the efficacy of a dual-pronged approach in enhancing solar cell efficiency and mitigating FF losses: reducing *R*_s_ and augmenting *R*_sh_. An increase in *R*_s_ stems from increased electrode and charge–transfer interface resistances, whereas a decrease in *R*_sh_ is tied to reduced functional layer coverage and the proliferation of pinholes. Despite the inherent challenge of fully eradicating internal resistance, these results underscore the imperative for experimental endeavors to craft electrodes with exceptional conductivity and minimal sheet resistance, alongside strategies to diminish interface charge–transfer resistance. The key determinants of this interfacial charge transfer resistance include the electrode–semiconductor contact resistance, the bulk resistance intrinsic to the semiconductor, barriers arising from interface band offsets, and recombination losses at the interface, exacerbated by impurities and defects [34]. To increase the overall performance of Sb_2_(S,Se)_3_ solar cells to unprecedented levels, concerted optimization endeavors targeting interface band alignment, interface recombination processes, and absorber defects are of paramount importance.

### 3.2. Optimization of the Energy Band Offset 

Despite significant advancements in enhancing the efficiency of Sb_2_(S,Se)_3_ solar cells through meticulous optimization of the internal resistance, resulting in a notable improvement in the FF, increasing the *V*_oc_ remains a formidable challenge. To overcome this obstacle, it is imperative to meticulously optimize the internal energy band arrangements within the solar cell architecture. Optimal energy level alignment can facilitate the seamless transportation of photogenerated electrons toward the front contact and holes toward the back contact, emanating from the absorber layer, thereby reducing the interface-induced recombination losses in Sb_2_(S,Se)_3_ solar cells. Establishing an ideal energy band offset (EBO) at the interfaces, which increases carrier transport properties and minimizes recombination losses, is of paramount importance [35]. Notably, the formation of EBO, a crucial aspect of Sb_2_(S,Se)_3_ solar cells, is closely linked to disparities in the electron affinity and energy gap across the material interface. Addressing these challenges through meticulous interface engineering is essential for mitigating recombination losses, enhancing *V*_oc_, and ultimately achieving higher overall efficiencies for these solar cells. The conduction band offset (CBO) existing at the junction of the ETL and absorber is fundamentally dictated by the disparity in electron affinities between these two layers ($\chi_{absorber}\;$ and $\chi_{ETL}$, respectively), as follows:

(7) $$CBO=\chi_{absorber}-{\;\chi }_{ETL}$$

The interface between materials can exhibit three distinct band alignment configurations, each characterized by the CBO. A negative CBO induces a steep cliff-like EBO, whereas a positive CBO yields a more pronounced spike-like EBO. When the CBO is zero, a flat band alignment is observed [36]. There is a general consensus among researchers that either a flat band alignment or a mild spike-like CBO is conducive to efficient charge transfer and the minimization of interface recombination losses [37]. 

**Figure 3:**
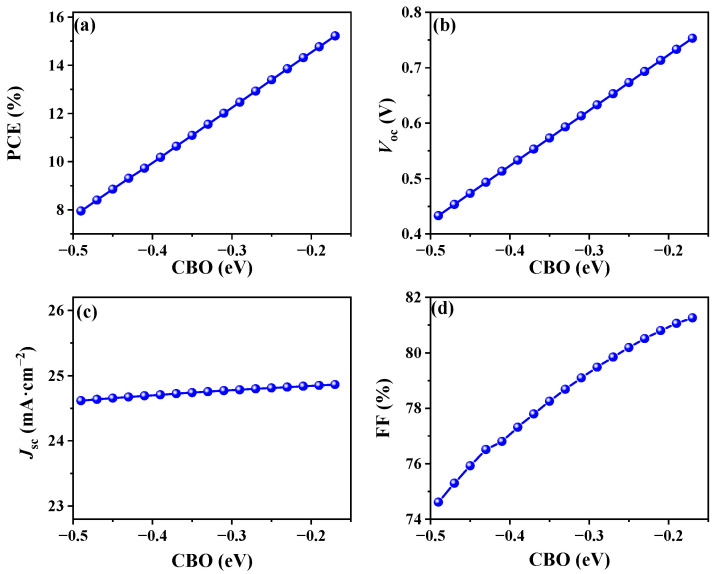
Simulation results for (**a**) PCE, (**b**) *V*_oc_, (**c**) *J*_sc_, and (**d**) FF with varied CBO from −0.49 to −0.17 eV (χ_ETL_ from 3.86 to 4.30 eV). Other parameters are based on Simulation 2.

Here, the band arrangements at the ETL/absorber interface were investigated by varying the χ_ETL_ from 4.3 eV to 3.7 eV, with the χ_absorber_ maintained at 3.81 eV. This range of χ_ETL_ corresponded to a CBO ranging from −0.49 eV to 0.19 eV, as depicted in Figure S1a. As the CBO shifted from negative to positive values, a distinct spike-like EBO emerged in the conduction band at the ETL/absorber interface, as expected. However, a remarkable observation was made at the FTO/ETL interface, where a considerably more prominent spike-like EBO manifested in the conduction band, as clearly shown in Figure S1b. This enhanced EBO brings additional obstacles to the transfer of electrons to the front electrode, ultimately hindering charge transfer with the increase in CBO. Therefore, when CBO decreases below −0.17 eV, the electrons can surmount the potential barrier at the FTO/ETL interface and reach the front electrode, thus avoiding computational convergence issues, as shown in Figure S1c. When $\chi_{ETL}$ was tuned from 4.3 eV to 3.98 eV (corresponding to a CBO from −0.49 eV to −0.17 eV), remarkable linear enhancements were observed in the PCE, *V*_oc_, and FF, whereas the *J*_sc_ remained relatively stable, as shown in Figure 3. This behavior originated from the reduction in cliff-like interfacial characteristics between the ETL and absorber, coupled with an enhanced built-in electric field at both the ETL/absorber and FTO/ETL interfaces (Figure S1d), resulting in reduced electron capture at the interface [38]. Thus, the Sb_2_(S,Se)_3_ solar cell reached its optimal performance at a CBO of −0.17 eV. 

Certainly, the refinement of the CBO at the ETL/absorber interface significantly enhances the transport of photogenerated electrons. Nevertheless, the employment of an appropriate HTL is of paramount importance in facilitating the migration of photogenerated holes toward the back contact while simultaneously impeding the undesirable reverse flow of electrons. Thus, the valence band offset (VBO) at the interface between the absorber and HTL critically governs the transport dynamics of photogenerated holes toward the back electrode in the Sb_2_(S,Se)_3_ absorber layer. The VBO value can be calculated as follows:

(8) $$\mathrm{VBO}=E_{v\text{\_}\mathrm{absorber}}-E_{v\text{\_}\mathrm{HTL}}=E_{g\text{\_}\mathrm{absorber}}+\chi_{absorber}-(E_{g\text{\_}\mathrm{HTL}}+\chi_{HTL})$$

**Figure 4:**
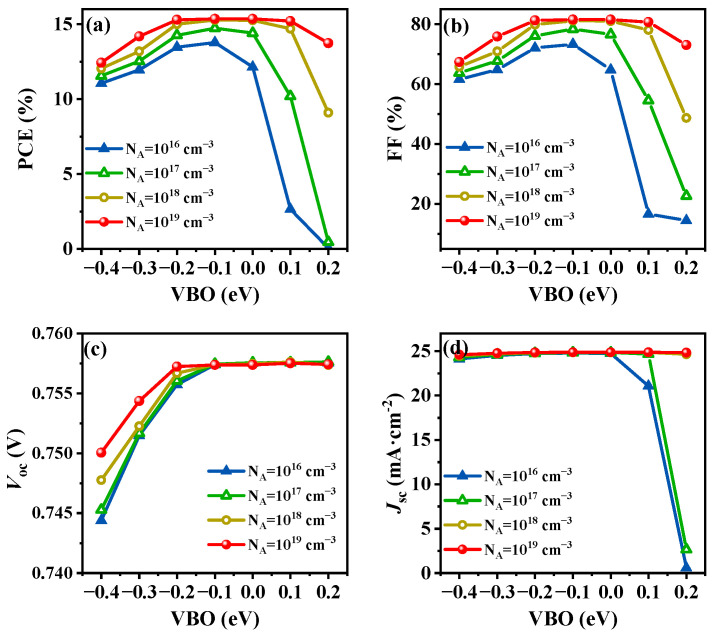
Simulation results for (**a**) PCE, (**b**) *V*_oc_, (**c**) *J*_sc_, and (**d**) FF as a function of varied VBO (−0.4 eV to 0.2 eV).

As shown in Figure 4a, the PCE demonstrates a consistent trend of an initial rise followed by a subsequent decline at all values of *N*_A_ in the HTL, when the VBO varies from −0.4 eV to 0.2 eV. This VBO variation corresponds to a range of E_v_HTL_ from 4.84 eV to 5.44 eV, with E_v_absorber_ fixed at 5.24 eV. Notably, across *N*_A_ values ranging from 10^16^ cm^−3^ to 10^19^ cm^−3^, the peak PCE occurs at a VBO of −0.1 eV. In Figure 4b, the FF exhibits a similar trend to that of the PCE. However, *V*_oc_ remains almost unchanged as the VBO increases to −0.1 eV, whereas *J*_sc_ initially remains constant and subsequently rapidly decreases after the VBO exceeds 0 eV (see Figure 4c,d). When the VBO exceeds 0 eV, the PCE, FF, and *J*_sc_ of the simulated device undergo rapid deterioration. This significant decline can be explained by the emergence of an energy barrier at the valence band of the absorber/HTL interface. This barrier considerably impedes the seamless flow of photogenerated holes toward the back electrode, leading to their accumulation at the interface. This accumulation, in turn, intensifies the surface recombination processes, further reducing the performance metrics of the device. When VBO is −0.1 eV (E_v_HTL_ = 5.14 eV) and *N*_A_ ranges between 10^18^ cm^−3^ and 10^19^ cm^−3^, the device exhibits optimal performance. 

As demonstrated by the simulation results, extreme positive or negative values of either the CBO or VBO adversely affect device performance. By optimizing the energy level alignment, we achieved an optimal CBO of −0.17 eV and VBO of −0.1 eV, which were applied in Simulation 3. Simulation 3 achieved a PCE of 15.31%, a *V*_oc_ of 0.76 V, a *J*_sc_ of 24.86 mA·cm^−2^, and an FF of 81.32%. Compared with Simulation 2, the device performance in Simulation 3 showed significant improvements, with a significant reduction of 15.19% in *V*_oc_ loss and a noteworthy 21.80% increase in the PCE, as detailed in Table 1. These results highlight the critical role of precise control over energy level alignment in optimizing photovoltaic device performance. To achieve this optimization while keeping the absorber material’s energy levels constant, the electron affinity of the ETL (CdS) needs to be reduced from 4.1 eV to 3.98 eV, and the electron affinity of the HTL (spiro-OMeTAD) needs to be reduced from 2.1 eV to 2.0 eV. In practice, it is necessary to optimize the conduction band energy levels of each functional layer material by adjusting doping elements or introducing specific functional groups to achieve better energy level matching, thereby constructing more efficient solar cell structures. 

### 3.3. Optimization of Interface Recombination

Even if optimal band alignment is achieved in the device structure, the potential effect of nonradiative recombination due to interface defects resulting from lattice mismatches between functional layers must be considered. Sb_2_(S,Se)_3_ possesses an orthorhombic crystal structure, while CdS typically has a hexagonal structure and spiro-OMeTAD adopts a triclinic structure [39]. The inherent structural incompatibility among the ETL, absorber, and HTL layers leads to the emergence of lattice mismatches at their junctions, fostering the development of interface defects that act as sites for nonradiative recombination. To simulate nonradiative recombination through the Shockley–Read–Hall (SRH) mechanism, the SRH recombination rate (*R*_SRH_) is considered. The *R*_SRH_ consists of three main components: bulk recombination (*R*_bulk_) within the absorber layer, interface recombination at the ETL/absorber interface (*R*_IF1_), and interface recombination at the absorber/HTL interface (*R*_IF2_) [39,40,41], as shown in Equation (9):

(9) $$R_{SRH}=R_{bulk}+R_{IF1}+R_{IF2}=\frac{(np-n_{i}^{2})\sigma_{n,p}\upsilon_{th}N_{t}}{n+p+2n_{i}cosh\frac{E_{t}-E_{i}}{k_{B}T}}$$

where *σ*_n_ and *σ*_p_ represent the capture cross-sections for electrons and holes, respectively; *υ*_th_ denotes the thermal velocity; and *n*_i_ and *E*_i_ represent the intrinsic density and energy level of materials, respectively. Additionally, *N*_t_ and *E*_t_ are the density and energy level of the defect states, respectively, and *S* represents the surface recombination velocity (defined as *S* = *σ*_n,p_υ_th_*N*_t_). 

**Figure 5:**
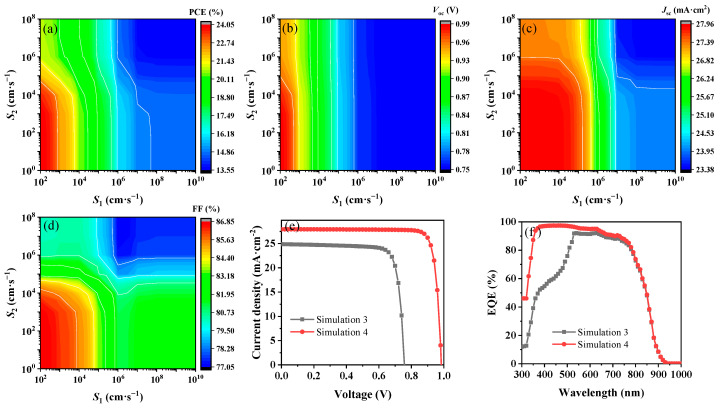
Simulated results for (**a**) PCE, (**b**) *V*_oc_, (**c**) *J*_sc_, and (**d**) FF as a function of varied *S*_1_ and *S*_2_. (**e**) *J–V* curves and (**f**) EQE spectra for Simulation 3 and Simulation 4.

The influence of the surface recombination velocity, namely *S*_1_ at the ETL/absorber interface and *S*_2_ at the absorber/HTL interface, on the performance parameters of Sb_2_(S,Se)_3_ solar cells is clearly illustrated in Figure 5a–d. When *S*_2_ is held constant and *S*_1_ varies between 10^6^ and 10^10^ cm·s^−1^, the *V*_oc_ remains below 0.80 V, with negligible changes in both the PCE and FF. However, when *S*_2_ is less than 10^4^ cm·s^−1^ and *S*_1_ decreases from 10^6^ to 10^2^ cm·s^−1^, the PCE gradually increases, ultimately exceeding 20%, and the *V*_oc_ progressively increases to nearly 1.0 V. These findings underscore the more significant impact of *S*_1_ than *S*_2_ on the *V*_oc_ and PCE of Sb_2_(S,Se)_3_ solar cells. This result is likely attributed to the presence of a p–n junction formed between the p-type Sb_2_(S,Se)_3_ absorber layer and the n-type CdS layer, which governs the behavior of holes as minority carriers within the Sb_2_(S,Se)_3_ thin film. In contrast, a relatively weaker p^+^–p junction is formed with p-type Spiro-OMeTAD. Thus, the optimization of the ETL/absorber interface through meticulous interface engineering can likely lead to a more effective increase in the performance of Sb_2_(S,Se)_3_ solar cells [42].

Notably, Simulation 4 with optimized parameters of 10^2^ cm·s^−1^ for *S*_1_ and 10^3^ cm·s^−1^ for *S*_2_ attained an impressive PCE of 23.75%, with excellent characteristics, such as an *n*_ID_ of 1.08, a *V*_oc_ loss of 0.45 V, and a negligible FF loss of 0.96%, as detailed in Table 1. As evident in Figure 5e, compared with simulation 3, optimizing the interface recombination velocities led to a notable increase in *V*_oc_ from 0.76 V to 0.98 V, *J*_sc_ from 24.86 mA·cm^−2^ to 27.93 mA·cm^−2^, and PCE from 15.31% to 23.75%. Figure 5f shows that this enhancement was attributed to the improved optical response of the device at wavelengths below 600 nm after reducing interface recombination. Reducing the trap density at the ETL/absorber interface from 10^14^ cm^−2^ to 10^10^ cm^−2^ or the capture cross-section from 10^−15^ cm^2^ to 10^−19^ cm^2^ is a significant challenge for practical experiments. Indeed, interface modification engineering can passivate surface defects and improve material smoothness and surface impurities, effectively reducing trap density and/or capturing cross-sections. For example, CdS thin films etched with N_2_H_4_ exhibit higher transmittance, smoother surfaces, and fewer Cd oxychlorides [12]. Additionally, adding insulating polymer layers can further reduce surface roughness and interface states, enhancing device performance [41].

### 3.4. Optimization of the Sb_2_(S,Se)_3_ Absorber’s Thickness and Bulk Defects 

**Figure 6:**
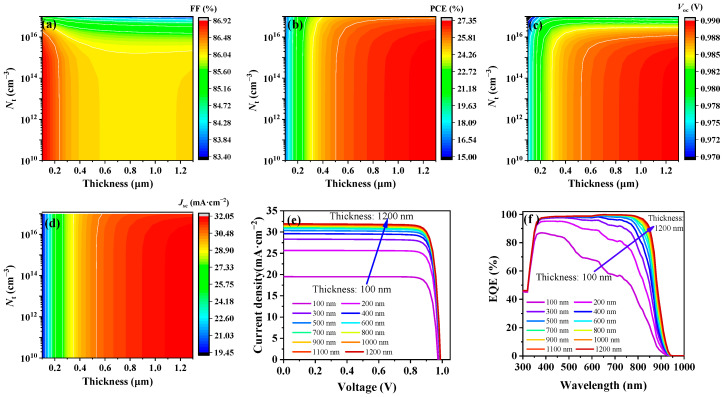
Simulated results for (**a**) FF, (**b**) PCE, (**c**) *V*_oc_, and (**d**) *J*_sc_ with varied *N*_t_ and thickness of the Sb_2_(S,Se)_3_ layer. (**e**) *J*–*V* curves and (**f**) EQE spectra with varied thickness of the Sb_2_(S,Se)_3_ layer.

The thickness of the Sb_2_(S,Se)_3_ absorber significantly affects various performance aspects of solar cells, including light absorption, carrier mobility, and recombination rates. Consequently, our subsequent simulations delved into the impact of varying the thickness and *N*_t_ of the absorber to pinpoint the optimal configuration for enhancing device performance. When *N*_t_ remained at 10^16^ cm^−3^ and the thickness remained below 600 nm, as evident from Figure 6a–d, there was a slight increase in *V*_oc_ and a slight decrease in FF with increasing thickness, whereas both the PCE and *J*_sc_ significantly increased. The increase in absorber thickness led to greater photon absorption, resulting in a greater number of photogenerated carriers and thus improving the *J*_sc_ and PCE. However, once the thickness exceeds 600 nm, the photon absorption approaches saturation and the PCE almost plateaus, as shown in Figure 6e. Furthermore, increasing the absorber thickness from 100 nm to 1200 nm led to a significant increase in the response of the EQE (see Figure 6f). Nevertheless, this increase in absorber thickness extended the transit distance for the charge carriers before collection and elevated the internal resistance of the device, consequently reducing the FF [42]. At a thickness of 600 nm, the FF stabilized at nearly 86.01%. Consequently, with an Sb_2_(S,Se)_3_ thickness of 600 nm and an initial *N*_t_ of 6.28 × 10^12^ cm^−3^, Simulation 5 achieved a PCE of 26.77%, an FF of 87.09%, a *V*_oc_ of 0.99 V, and a *J*_sc_ of 30.93 mA·cm^−2^, as summarized in Table 1.

### 3.5. Optimization of the Work Function at the Back Contact Layer

**Figure 7:**
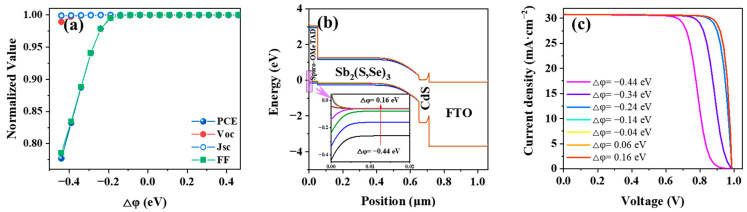
(**a**) The influence of Δφ on device performance, (**b**) the energy band diagrams, and (**c**) *J*–*V* curses with varied Δφ from −0.44 eV to 0.16 eV.

The variation in the work function of the back contact (φ_BC_) directly impacts the energy band structure at the rear of solar cells, thereby impacting the collection of photogenerated carriers. We conducted a study to examine the influence of φ_BC_ on the performance of Sb_2_(S,Se)_3_ solar cells, with a specific focus on the difference Δφ (referred to as E_v_HTL_ − φ_BC_). When Δφ ranges from −0.44 eV to 0.46 eV, φ_BC_ shifts within a range of 4.74 eV to 5.60 eV. Figure 7a reveals a consistent increasing trend in both the PCE and FF, reaching their maximum values at −0.14 eV. Beyond this Δφ value, the PCE and FF remain stable. The downward curvature in the valence band and the characteristic S-shaped curve in the current–voltage characteristics when Δφ falls below −0.14 eV, as depicted in Figure 7b,c, clarify this phenomenon. The varying energy levels across the HTL/back contact interface, causing the emergence of a Schottky barrier within the Δφ range of −0.44 eV to −0.14 eV, are responsible for impeding the unhampered flow of holes from the HTL toward the back electrode, as shown in Figure 7b. Notably, when Δφ approaches or exceeds −0.14 eV, the barrier gradually diminishes, allowing for improved hole transport and reduced resistance at the HTL/back contact interface, and all device parameters are saturated. In other words, when φ_BC_ exceeds 5.04 eV, the back electrode and HTL form a good ohmic contact conducive to hole collection. The back contact employed in Simulation 5 is gold (φ_BC_ = 5.1 eV), which falls within the optimized range, and the back contact of the Sb_2_(S,Se)_3_ solar cell can be unchanged.

### 3.6. Final Optimized Sb_2_(S,Se)_3_ Solar Cell Performance

After the above optimization analysis, the final values are as follow: the thickness of Sb_2_(S,Se)_3_ is 600 nm, and the electron affinities of the ETL (CdS) and HTL (spiro-OMeTAD) are optimized to 3.98 eV and 2.04 eV, respectively; the interface recombination velocities of ETL/absorber and absorber/HTL are optimized at 10^2^ cm·s^−1^ and 10^3^ cm·s^−1^, respectively. Additionally, the back electrode with a work function greater than 5.04 eV (e.g., Au~5.1 eV) can form a better ohmic contact with HTL, and the series and shunt resistances of the device are optimized to 0 and 10^5^ Ω·cm^2^, respectively. As shown in Table 1, the final optimized device (simulation 5) has achieved excellent performance parameters: *V*_oc_ = 0.99 V, *J*_sc_ = 30.93 mA·cm^−2^, FF = 87.09%, and PCE = 26.77%. Compared with other simulation results presented in Table S4, the performance of our simulated Sb_2_(S,Se)_3_ solar cell is superior, due to the effective optimization path chosen based on *V*_oc_ and FF losses to enhance the device structure.

## 4. Improvement in the PCE of the Sb_2_(S,Se)_3_ Solar Cells

Based on the preceding simulation outcomes, a two-phased approach is proposed to increase the efficiency of Sb_2_(S,Se)_3_ solar cells. Initially, an efficiency benchmark of up to 15% was achieved, and subsequently, the goal was to surpass the 20% efficiency threshold.

**Approaching 15% efficiency**. Although the lowest *V*_oc_ loss reported in Sb_2_(S,Se)_3_ solar cells has an *E*_g_ of 1.46 eV [3], the *V*_oc_ value remains at approximately 0.69 V, which is notably far lower than the S–Q limit *V*_oc_ of 1.19 V. This discrepancy is commonly observed in Sb_2_(S,Se)_3_ solar cells with PCEs surpassing 10% [3,4,11,13,14,15,43,44,45], highlighting a persistent challenge in their performance improvement [26]. To overcome this challenge, feasible methods for enhancing carrier transport properties, minimizing recombination losses, and improving the internal resistance of Sb_2_(S,Se)_3_ solar cells include the following:

(1) **Regulation of the absorption film to reduce the internal resistance.** The Sb_2_(S,Se)_3_ compound possesses a unique quasi-1D ribbon architecture characterized by [Sb_4_S(e)_6_]_n_ ribbons [46]. This structural peculiarity engenders favorable grain morphology and anisotropic carrier mobility, most notably along the [*hk*1] direction. This specific transport pathway underscores the prospect of significantly elevated device performance, particularly in comparison with transport along the [*hk*0] direction. Different synthesis/fabrication strategies have been used to develop [*hk*1]-oriented Sb_2_(S,Se)_3_ films. A highly efficient method lies in rapid hydrothermal deposition, which kinetically promotes the growth of [221]-oriented crystals [15]. Additionally, during synthesis processes such as close space sublimation (CSS) [47], rapid thermal evaporation (RTE) [45], and vapor transport deposition (VTD) [48], carefully balancing the temperature between the evaporation source and the substrate has been shown to be crucial for promoting [*hk*1] orientation and enhancing the crystallinity of Sb_2_(S,Se)_3_ films. High-quality crystals, characterized by low defect density, extended carrier lifetimes, elevated electron and hole mobilities, and uniform crystal structures, play a pivotal role in reducing internal resistance and optimizing carrier transport in solar cells.
(2) **Band alignment absorber layer and charge-transport layers.** To fully unlock the potential of Sb_2_(S,Se)_3_ solar cells in terms of the PCE, the optimization of the carrier transport path has emerged as a paramount step forward in advancing their performance. This can be achieved through careful adjustment of CBO at the ETL/absorber interface and VBO at the absorber/HTL interfaces [49]. The proper alignment of the energy bands between the absorber layer and charge–transport layers is essential for minimizing energy-level mismatches and reducing the interface energy barriers. Optimizing the interface energy barrier involves using ETLs and HTLs with minimal defects or employing doping strategies to mitigate band mismatch. For example, nitrogen-containing functional groups in ethylenediamine (EDA) can coordinate with CdS, and shift the Fermi level of EDA–CdS toward the conduction band [50]. Alternatively, in situ oxygen doping of CdS films results in a lower conduction band level compared to that of control CdS films [51]. Various configurations of CdS-based ETLs such as SnO_2_/CdS [25,52], TiO_2_/CdS [53,54], ZnO/CdS [55], and Zn(O,S)/CdS [56] ensure well-matched band alignment, thereby facilitating efficient charge transport in Sb_2_(S,Se)_3_ solar cells.

**Exceeding 20% efficiency**. Reducing SRH recombination is a critical aspect of improving the performance of Sb_2_(S,Se)_3_ solar cells. By implementing appropriate passivation strategies and surface treatments, such as postselenization [57,58] and advanced interfacial engineering [59,60], it is feasible to significantly diminish the defect density within a material. This reduction not only mitigates the entrapment of charge carriers but also increases their lifetime. Achieving an efficiency of more than 20% in Sb_2_(S,Se)_3_ solar cells requires suitable methods, including the following:

(1) **Regulation of the interface recombination.** Annealing and interface treatments are essential for rectifying instability, smoothing out roughness, and enhancing conductivity within ETLs. This helps minimize the defect density and lower carrier recombination at the ETL/absorber junction. For example, oxygen-doped cadmium sulfide (CdS:O) facilitates the tailoring of (Sb_4_S(e)_6_)_n_ ribbons to the (221)-textured orientation [61], CdCl_2_-modified CdS films passivate surface defects [62], and KCl-treated CdS films promote absorption layer growth [10]. Furthermore, enhancing the p-type characteristics of HTL materials through doping or developing low-cost and efficient alternatives is crucial for increasing hole mobility and improving material conductivity, such as high-mobility and work-function NiO_x_ [63,64], excellent near-infrared-absorption PbS colloidal quantum dots [7,65], and high-hole-mobility and high-stability CuSCN [66]. These alternative approaches hold significant potential in increasing the performance of solar cells, primarily through the enhancement of hole mobility and the mitigation of interface recombination.
(2) **Balance of the relationship between the thickness of the absorber and bulk defects.** Both theoretical calculations [67,68,69] and experimental measurements [3,70] have demonstrated the complex defect mechanisms in Sb_2_(S,Se)_3_. The thickness of the absorber layer influences both photon absorption and carrier transport [71]. If the absorber layer is too thin, insufficient photon absorption occurs, and pinhole formation increases, thereby resulting in a low *J*_sc_. However, during the actual preparation process, overly thick layers increase photon absorption while also increasing the series resistance and carrier transport distance, thereby intensifying carrier recombination. The thickness of the absorber layer can be controlled by adjusting the synthesis/fabrication time and growth rate. Furthermore, the absorber bulk defects are reduced through empirical methods such as adjusting the Se/S atomic ratio [15,72], postselenization treatments [73], seeding materials [74,75], or additive engineering [4,76].

## 5. Conclusions

In summary, a numerical model of Sb_2_(S,Se)_3_ solar cells was developed utilizing the SCAPS software (version 3.3.10; University of Gent, Belgium) package and validated against experimental data from the literature. This model was used to investigate the factors limiting *V*_oc_ and FF at various stages of Sb_2_(S,Se)_3_ solar cell development, and effective strategies were identified to increase the PCE of these cells. The simulation results indicated an improvement in the PCE from 10.72% to 12.57%, as well as a significant increase in the FF from 68.35% to 79.55% after addressing the limiting mechanisms related to *R*_s_ and *R*_sh_. Further adjustments in the CBO at the ETL/absorber and VBO at the absorber/HTL interfaces led to an increase in the PCE from 12.57% to 15.31%, along with a notable 18.75% improvement in *V*_oc_. These results highlight the importance of reducing the internal resistance to enhance the FF and show the importance of optimal energy level arrangements for increasing *V*_oc_. 

The subsequent optimization of interface recombination led to a notable increase in the overall performance of Sb_2_(S,Se)_3_ solar cells, resulting in a remarkable PCE exceeding 20%. This was accompanied by a decrease in the *V*_oc_ loss to 0.45 V, an increase in the *J*_sc_ to 27.93 mA·cm^−2^, and a reduction in the FF loss to 0.96%. These results highlight the crucial role of suppressing interface SRH recombination, particularly at the ETL/absorber interface, in improving the overall performance of devices. By striking a balance between the absorber thickness and the bulk defects, the *J*_sc_ increased to over 30 mA·cm^−2^ at a thickness of 600 nm, consequently increasing the PCE to an impressive 26.77%. 

This numerical analysis proposes an optimization roadmap aimed at reducing *V*_oc_ and FF losses, thereby successfully enhancing the efficiency of Sb_2_(S,Se)_3_ solar cells. While numerical simulations cannot completely replace experimental work, they undoubtedly provide significant guidance for future experimental efforts to improve the efficiency of Sb_2_(S,Se)_3_ solar cells, helping to avoid blind experimental exploration and thus saving valuable time and resources. Furthermore, this study proposes a two-stage strategy based on simulation results to further boost the efficiency of Sb_2_(S,Se)_3_ solar cells. We hope these findings can also be extended to other solar materials, such as Sb_2_Se_3_ and Sb_2_S_3_, thereby broadening their applicability beyond current boundaries.

## Figures and Tables

**Table 1:** Photovoltaic parameters in five simulated devices.

	*V*_oc_ (V)	FF (%)	*V*_oc_ Losst ^(a)^ (V)	FF Loss ^(b)^ (%)	*n*_ID_	*J*_sc_ (mA·cm^−2^)	PCE (%)
Simulation 1	0.64	68.35	0.79	10.79	1.39	24.63	10.72
Simulation 2	0.64	79.55	0.79	2.80	1.10	24.79	12.57
Simulation 3	0.76	81.31	0.67	3.02	1.11	24.86	15.31
Simulation 4	0.98	86.34	0.45	0.96	1.08	27.93	23.75
Simulation 5	0.99	87.09	0.44	0.31	1.08	30.93	26.77

^(a)^ *V*_oc_ loss is equal to *E*_g_/*q* − *V*_oc_, where *E*_g_ represents the bandgap of the active absorber and *q* is the elementary charge. ^(b)^ FF loss is equal to FF_m_ − FF, where FF_m_ denotes the maximum achievable FF across varying *V*_oc_ values in a single solar cell.

## Data Availability

Data will be available upon valid request.

## Supplementary Materials

The following supporting information can be downloaded at: https://www.mdpi.com/article/10.3390/nano14171433/s1, Figure S1: (a) Energy band diagram of Sb_2_(S,Se)_3_ solar cells under different χ_ETL_, (b) The conduction band diagram of the dashed circle in (a), (c) The conduction band diagram at CBO = −0.17 eV, (d) The variation of built-in electric field at ETL/absorber and FTO/ETL interfaces with CBO varied from −0.49 eV to −0.17 eV; Table S1: Simulation parameters applied to different layers within the solar cell model; Table S2: Simulation parameters for bulk defects in Sb_2_(S,Se)_3_; Table S3: Defect parameters at the interfaces of CdS/Sb_2_(S,Se)_3_ and Sb_2_(S,Se)_3_/spiro-OMeTAD; Table S4: Performance Comparison of Sb_2_(S,Se)_3_ Solar Cells: This Work vs. Published Studies. References [3,4,9,18,19,20,21,22,23,24,25,39,77,78,79,80,81,82] are cited in the Supplementary Materials.

## References

[1] Nicolás-Marín M.M., González-Castillo J.R., Vigil-Galán O., Courel M. (2022). The state of the art of Sb_2_(S,Se)_3_ thin film solar cells: Current progress and future prospect. J. Phys. D Appl. Phys..

[2] Wang J., Li K., Tang J., Chen C. (2023). A Perspective of Antimony Chalcogenide Photovoltaics toward Commercialization. Sol. RRL.

[3] Dong J., Liu H., Ding L., Che B., Xiao P., Cao Z., Liu Y., Lou L., Tang R., Luo J. (2023). Lowest Open-Circuit Voltage Deficit Achievement to Attain High Efficient Antimony Selenosulfide Solar Cells. Adv. Funct. Mater..

[4] Chen X., Che B., Zhao Y., Wang S., Li H., Gong J., Chen G., Chen T., Xiao X., Li J. (2023). Solvent-Assisted Hydrothermal Deposition Approach for Highly-Efficient Sb_2_(S,Se)_3_ Thin-Film Solar Cells. Adv. Energy Mater..

[5] Rühle S. (2016). Tabulated values of the Shockley–Queisser limit for single junction solar cells. Sol. Energy.

[6] Ding C., Wang D., Liu D., Li H., Li Y., Hayase S., Sogabe T., Masuda T., Zhou Y., Yao Y. (2022). Over 15% Efficiency PbS Quantum-Dot Solar Cells by Synergistic Effects of Three Interface Engineering: Reducing Nonradiative Recombination and Balancing Charge Carrier Extraction. Adv. Energy Mater..

[7] Chen C., Wang L., Gao L., Nam D., Li D., Li K., Zhao Y., Ge C., Cheong H., Liu H. (2017). 6.5% Certified Efficiency Sb_2_Se_3_ Solar Cells Using PbS Colloidal Quantum Dot Film as Hole-Transporting Layer. ACS Energy Lett..

[8] Bertens K., Fan J.Z., Biondi M., Rasouli A.S., Lee S., Li P., Sun B., Hoogland S., de Arquer F.P.G., Lu Z.-H. (2020). Colloidal Quantum Dot Solar Cell Band Alignment using Two-Step Ionic Doping. ACS Mater. Lett..

[9] Zhou R., Li X., Wan L., Niu H., Wang H., Yang X., Wang X., Hou J., Xu J., Xu B. (2023). Bulk Heterojunction Antimony Selenosulfide Thin-Film Solar Cells with Efficient Charge Extraction and Suppressed Recombination. Adv. Funct. Mater..

[10] Liu A., Tang R., Huang L., Xiao P., Dong Y., Zhu C., Wang H., Hu L., Chen T. (2023). KCl Treatment of CdS Electron-Transporting Layer for Improved Performance of Sb_2_(S,Se)_3_ Solar Cells. ACS Appl. Mater. Interfaces.

[11] Dong J., Liu H., Cao Z., Liu Y., Bai Y., Chen M., Liu B., Wu L., Luo J., Zhang Y. (2022). Low-Cost Antimony Selenosulfide with Tunable Bandgap for Highly Efficient Solar Cells. Small.

[12] Dong H., Zhang L., Che B., Xiao P., Wang H., Zhu C., Chen T. (2022). Hydrothermal Deposition of Antimony Selenosulfide on Titanium Oxide Nanoparticle Films for Cadmium-Free Solar Cells. ACS Appl. Opt. Mater..

[13] Li J., Zhao Y., Li C., Wang S., Chen X., Gong J., Wang X., Xiao X. (2022). Hydrazine Hydrate-Induced Surface Modification of CdS Electron Transport Layer Enables 10.30%-Efficient Sb_2_(S,Se)_3_ Planar Solar Cells. Adv. Sci..

[14] Wang X., Tang R., Jiang C., Lian W., Ju H., Jiang G., Li Z., Zhu C., Chen T. (2020). Manipulating the Electrical Properties of Sb_2_(S,Se)_3_ Film for High-Efficiency Solar Cell. Adv. Energy Mater..

[15] Tang R., Wang X., Lian W., Huang J., Wei Q., Huang M., Yin Y., Jiang C., Yang S., Xing G. (2020). Hydrothermal deposition of antimony selenosulfide thin films enables solar cells with 10% efficiency. Nat. Energy.

[16] Decock K., Zabierowski P., Burgelman M. (2012). Modeling metastabilities in chalcopyrite-based thin film solar cells. J. Appl. Phys..

[17] Burgelman M., Nollet P., Degrave S. (2000). Modelling polycrystalline semiconductor solar cells. Thin Solid Films.

[18] Chen Y., Wang Y., Wang R., Hu X., Tao J., Weng G.-E., Zhao C., Chen S., Zhu Z., Chu J. (2020). Importance of Interfacial Passivation in the High Efficiency of Sb_2_Se_3_ Thin-Film Solar Cells: Numerical Evidence. ACS Appl. Energy Mater..

[19] Hajjiah A., Gamal M., Kandas I., Gorji N.E., Shehata N. (2022). DFT and AMPS-1D simulation analysis of all-perovskite solar cells based on CsPbI_3_/FAPbI_3_ bilayer structure. Sol. Energy Mater. Sol. Cells.

[20] Xing Y., Guo H., Liu J., Zhang S., Qiu J., Yuan N., Ding J. (2022). High-efficiency Sb_2_(S,Se)_3_ solar cells with MoO_3_ as a hole-transport layer. J. Alloy. Compd..

[21] Ngoupo A.T., Ouédraogo S., Zougmoré F., Ndjaka J. (2021). Numerical analysis of ultrathin Sb_2_Se_3_-based solar cells by SCAPS-1D numerical simulator device. Chin. J. Phys..

[22] Zhou B., Yin X., Zhang J., Zeng G., Li B., Zhang J., Feng L. (2020). Numerical simulation of an innovative high efficiency solar cell with CdTe/Si composite absorption layer. Opt. Mater..

[23] Khadir A. (2023). Sb_2_(S,Se)_3_-Based Thin Film Solar Cells: Numerical Investigation. Acta Phys. Pol. A.

[24] Mamta, Maurya K., Singh V. (2021). Sb_2_Se_3_ versus Sb_2_S_3_ solar cell: A numerical simulation. Sol. Energy.

[25] Mao X., Bian M., Wang C., Zhou R., Wan L., Zhang Z., Zhu J., Chen W., Shi C., Xu B. (2022). Ultrathin SnO_2_ Buffer Layer Aids in Interface and Band Engineering for Sb_2_(S,Se)_3_ Solar Cells with over 8% Efficiency. ACS Appl. Energy Mater..

[26] Chen C., Tang J. (2020). Open-Circuit Voltage Loss of Antimony Chalcogenide Solar Cells: Status, Origin, and Possible Solutions. ACS Energy Lett..

[27] Shockley W., Queisser H.J. (1961). Detailed Balance Limit of Efficiency of p-n Junction Solar Cells. J. Appl. Phys..

[28] Green M.A. (1981). Solar cell fill factors: General graph and empirical expressions. Solid-State Electron..

[29] Qi B., Wang J. (2013). Fill factor in organic solar cells. Phys. Chem. Chem. Phys..

[30] Shi J., Zhao C., Yuan J. (2023). Achieving High Fill Factor in Efficient P-i-N Perovskite Solar Cells. Small.

[31] Kim H.D., Ohkita H. (2017). Potential Improvement in Fill Factor of Lead-Halide Perovskite Solar Cells. Sol. RRL.

[32] Ebon I.R., Ali H., Haque D., Islam A.Z.M.T. (2023). Computational investigation towards highly efficient Sb_2_Se_3_ based solar cell with a thin WSe2 BSF layer. Eng. Res. Express.

[33] Al Ahmed S.R., Sunny A., Rahman S. (2020). Performance enhancement of Sb_2_Se_3_ solar cell using a back surface field layer: A numerical simulation approach. Sol. Energy Mater. Sol. Cells.

[34] Mehmood S., Xia Y., Qu F., He M. (2023). Investigating the Performance of Efficient and Stable Planer Perovskite Solar Cell with an Effective Inorganic Carrier Transport Layer Using SCAPS-1D Simulation. Energies.

[35] Salem M.S., Shaker A., Abouelatta M., Alanazi A., Al-Dhlan K.A., Almurayziq T.S. (2022). Numerical analysis of hole transport layer-free antimony selenide solar cells: Possible routes for efficiency promotion. Opt. Mater..

[36] Ding C., Zhang Y., Liu F., Kitabatake Y., Hayase S., Toyoda T., Yoshino K., Minemoto T., Katayama K., Shen Q. (2018). Effect of the conduction band offset on interfacial recombination behavior of the planar perovskite solar cells. Nano Energy.

[37] Barthwal S., Gupta R., Kumar A., Ramesh K., Pathak S., Karak S. (2023). Band offset engineering in antimony sulfide (Sb_2_S_3_) solar cells, using SCAPS simulation: A route toward PCE > 10%. Optik.

[38] Chen H., Li Z.-Q., Sun B., Feng X.-D. (2021). Towards high-efficiency planar heterojunction antimony sulfide solar cells. Opt. Mater..

[39] Ayala-Mató F., Vigil-Galán O., Nicolás-Marín M.M., Courel M. (2021). Study of loss mechanisms on Sb_2_(S_1−x_Se_x_)_3_ solar cell with n–i–p structure: Toward an efficiency promotion. Appl. Phys. Lett..

[40] Wang D., Li Y., Yang Y., Hayase S., Wu H., Wang R., Ding C., Shen Q. (2023). How to minimize voltage and fill factor losses to achieve over 20% efficiency lead chalcogenide quantum dot solar cells: Strategies expected through numerical simulation. Appl. Energy.

[41] Wang A., Wang X., Chen Y. (2022). Investigation of the fundamental working mechanism for high-performance Sb_2_(S_1−x_Se_x_)_3_ solar cells. Eur. Phys. J. Plus.

[42] Li Z.-Q., Ni M., Feng X.-D. (2019). Simulation of the Sb_2_Se_3_ solar cell with a hole transport layer. Mater. Res. Express.

[43] Zhao Y., Wang S., Jiang C., Li C., Xiao P., Tang R., Gong J., Chen G., Chen T., Li J. (2021). Regulating Energy Band Alignment via Alkaline Metal Fluoride Assisted Solution Post-Treatment Enabling Sb_2_(S,Se)_3_ Solar Cells with 10.7% Efficiency. Adv. Energy Mater..

[44] Che B., Cai Z., Xiao P., Li G., Huang Y., Tang R., Zhu C., Yang S., Chen T. (2022). Thermally Driven Point Defect Transformation in Antimony Selenosulfide Photovoltaic Materials. Adv. Mater..

[45] Gao J., Che B., Cai H., Xiao P., Zhang L., Cai Z., Zhu C., Tang R., Chen T. (2023). Single-source thermal evaporation converts anion controllable Sb_2_(S,Se)_3_ film for fabricating high-efficiency solar cell. Sci. China Mater..

[46] Huang M., Cai Z., Chen S. (2020). Quasi-one-dimensional Sb_2_(S,Se)_3_ alloys as bandgap-tunable and defect-tolerant photocatalytic semiconductors. J. Chem. Phys..

[47] Li K., Xie Y., Zhou B., Li X., Gao F., Xiong X., Li B., Zeng G., Ghali M. (2021). Fabrication of closed-space sublimation Sb_2_(S1-xSex)_3_ thin-film based on a single mixed powder source for photovoltaic application. Opt. Mater..

[48] Zhang L., Bai X., Cui X., Zhang M. (2023). Vapor transport deposited Sb_2_(S,Se)_3_ thin film: Effect of deposition temperature and Sb_2_S_3_/Sb_2_Se_3_ mass ratio. J. Cryst. Growth.

[49] Zhao Y., Chen X., Li J., Xiao X. (2023). A Review of Carrier Transport in High-Efficiency Sb_2_(S,Se)_3_ Solar Cells. Sol. RRL.

[50] Gu Y., Liang W., Che Y., Cai Z., Xiao P., Yang J., Zang R., Wang H., Wu X., Chen T. (2023). Solvent Annealing Enabling Reconstruction of Cadmium Sulfide Film for Improved Heterojunction Quality and Photovoltaic Performance of Antimony Selenosulfide Solar Cells. Adv. Funct. Mater..

[51] Li K., Cai Z., Yang J., Wang H., Zhang L., Tang R., Zhu C., Chen T. (2023). Molecular Beam Epitaxy Deposition of In Situ O-Doped CdS Films for Highly Efficient Sb_2_(S,Se)_3_ Solar Cells. Adv. Funct. Mater..

[52] Shi X., Zhang F., Dai S., Zeng P., Qu J., Song J. (2022). Nanorod-textured Sb_2_(S,Se)_3_ bilayer with enhanced light harvesting and accelerated charge extraction for high-efficiency Sb_2_(S,Se)_3_ solar cells. Chem. Eng. J..

[53] Wang W., Wang X., Chen G., Chen B., Cai H., Chen T., Chen S., Huang Z., Zhu C., Zhang Y. (2018). Promising Sb_2_(S,Se)_3_ Solar Cells with High Open Voltage by Application of a TiO_2_/CdS Double Buffer Layer. Sol. RRL.

[54] Wu C., Jiang C., Wang X., Ding H., Ju H., Zhang L., Chen T., Zhu C. (2018). Interfacial Engineering by Indium-Doped CdS for High Efficiency Solution Processed Sb_2_(S_1−x_Se_x_)_3_ Solar Cells. ACS Appl. Mater. Interfaces.

[55] Ishaq M., Deng H., Yuan S., Zhang H., Khan J., Farooq U., Song H., Tang J. (2018). Efficient Double Buffer Layer Sb_2_(Se_x_S_1−x_)_3_ Thin Film Solar Cell Via Single Source Evaporation. Sol. RRL.

[56] Zhao Y., Li C., Niu J., Zhi Z., Chen G., Gong J., Li J., Xiao X. (2021). Zinc-based electron transport materials for over 9.6%-efficient S-rich Sb_2_(S,Se)_3_ solar cells. J. Mater. Chem. A.

[57] Rijal S., Adhikari A., Awni R.A., Xiao C., Li D.-B., Dokken B., Ellingson A., Flores E., Bista S.S., Pokhrel D. (2022). Post-Annealing Treatment on Hydrothermally Grown Antimony Sulfoselenide Thin Films for Efficient Solar Cells. Sol. RRL.

[58] Lin Y.-C., Chang C.-H., Hung Y.-J. (2023). Bandgap grading via sputtering and post-selenization using SeS_2_ powder enabling Sb_2_(S,Se)_3_ solar cells with 7.1% efficiency. Sol. Energy Mater. Sol. Cells.

[59] Wang C., Li D., Mao X., Wan L., Cheng Z., Zhu J., Hoye R.L.Z., Zhou R. (2023). Interfacial defect healing of In_2_S_3_/Sb_2_(S,Se)_3_ heterojunction solar cells with a novel wide-bandgap InOCl passivator. J. Mater. Chem. A.

[60] Pan X., Pan Y., Wang L., Zhao C., Hu X., Jiang J., Yang B., Chen S., Yang P., Chu J. (2023). Interfacial engineering by applying double CdS structure electron transport layer for high-performance Sb_2_(S,Se)_3_ solar cells. Ceram. Int..

[61] Dong J., Liu Y., Wang Z., Zhang Y. (2021). Boosting V_OC_ of antimony chalcogenide solar cells: A review on interfaces and defects. Nano Sel..

[62] Han X., Shi C., Huang Y., Lv K., Chen W., Guo F., Wang Y. (2023). Precise Preparation of CdS Thin Films for Efficient Sb_2_S_3_–ySey Thin-Film Solar Cells. ACS Appl. Energy Mater..

[63] Huang S., Xing Y., Zhu H., Zhang T., Geng K., Yang Y., Zhang H., Gu Q., Qiu J., Jiang S. (2024). Enhancement in the efficiency of Sb_2_(S,Se)_3_ thin-film solar cells with spin-coating NiOx as the hole transport layer. J. Mater. Chem. C.

[64] Li J., Hu X., Zheng X., Gao Z., Wang S., Liu Y., Wang C., Shao W., Fang G. (2023). Stability and Efficiency Enhancement of Antimony Selenosulfide Solar Cells with Inorganic SnS-Modified Nickel Oxide Hole Transport Materials. Energy Technol..

[65] Wei Y., Ding C., Shi G., Bi H., Li Y., Li H., Liu D., Yang Y., Wang D., Chen S. (2024). Stronger Coupling of Quantum Dots in Hole Transport Layer Through Intermediate Ligand Exchange to Enhance the Efficiency of PbS Quantum Dot Solar Cells. Small Methods.

[66] Li K., Wang S., Chen C., Kondrotas R., Hu M., Lu S., Wang C., Chen W., Tang J. (2019). 7.5% n–i–p Sb_2_Se_3_ solar cells with CuSCN as a hole-transport layer. J. Mater. Chem. A.

[67] Zhang B., Qian X. (2022). Competing Superior Electronic Structure and Complex Defect Chemistry in Quasi-One-Dimensional Antimony Chalcogenide Photovoltaic Absorbers. ACS Appl. Energy Mater..

[68] Savory C.N., Scanlon D.O. (2019). The complex defect chemistry of antimony selenide. J. Mater. Chem. A.

[69] Huang M., Xu P., Han D., Tang J., Chen S. (2019). Complicated and Unconventional Defect Properties of the Quasi-One-Dimensional Photovoltaic Semiconductor Sb_2_Se_3_. ACS Appl. Mater. Interfaces.

[70] Huang Y., Tang R., Wang G., Li G., Che B., Wang Y., Lian W., Zhu C., Chen T. (2022). Chemical insight into the hydrothermal deposition of Sb_2_(S,Se)_3_ towards delicate microstructure engineering. J. Mater. Chem. A.

[71] Wu M., Han N., Chen Y., Zeng H., Li X. (2023). Performance investigation of antimony chalcogenide based thin film solar cells via SCAPS simulation. Optik.

[72] Wang R., Qin D., Zheng S., Weng G., Hu X., Tao J., Chu J., Akiyama H., Chen S. (2023). Influence of S-content ratios on the defect properties of Sb_2_(S, Se1–)_3_ thin-film solar cells. Sol. Energy Mater. Sol. Cells.

[73] Chen G.-J., Tang R., Chen S., Zheng Z.-H., Su Z.-H., Ma H.-L., Zhang X.-H., Fan P., Liang G.-X. (2022). Crystal Growth Promotion and Defect Passivation by Hydrothermal and Selenized Deposition for Substrate-Structured Antimony Selenosulfide Solar Cells. ACS Appl. Mater. Interfaces.

[74] Zhang L., Lian W., Zhao X., Yin Y., Chen T., Zhu C. (2020). Sb_2_S_3_ Seed-Mediated Growth of Low-Defect Sb_2_S_3_ on a TiO_2_ Substrate for Efficient Solar Cells. ACS Appl. Energy Mater..

[75] Amin A., Li D., Duan X., Vijayaraghavan S.N., Menon H.G., Wall J., Weaver M., Cheng M.M., Zheng Y., Li L. (2022). Enhanced Efficiency and Stability in Sb_2_S_3_ Seed Layer Buffered Sb_2_Se_3_ Solar Cells. Adv. Mater. Interfaces.

[76] Baron-Jaimes A., Ortiz-Soto K.A., Millán-Franco M.A., Gamboa R.A.M., Rincón M.E., Jaramillo-Quintero O.A. (2023). Additive engineering by tetrabutylammonium iodide for antimony selenosulfide solar cells. J. Phys. D Appl. Phys..

[77] Mamta, Kumari R., Maurya K., Singh V. (2022). Sb_2_(S,Se)_3_-based photovoltaic cell with MoS2 as a hole transport layer: A numerical investigation. Mater. Today Sustain..

[78] Nicolás-Marín M., Ayala-Mato F., Vigil-Galán O., Courel M. (2021). Simulation analysis of Cd_1−x_Zn_x_S/Sb_2_(Se_1−x_S_x_)_3_ solar cells with n-i-p structure. Sol. Energy.

[79] Nicolás-Marín M.M., Vigil-Galán O., Ayala-Mato F., Courel M. (2022). Analysis of Hole Transport Layer and Electron Transport Layer Materials in the Efficiency Improvement of Sb_2_(Se_1−*x*_S*_x_*)_3_ Solar Cell. Phys. Status Solidi (B).

[80] Sekar K., Mayarambakam S. (2023). Effect of Annealed and Non-Annealed Inorganic MnS Hole-Transport Layer for Efficient Sb_2_(S,Se)_3_ Solar Cells: A Theoretical Justification. Phys. Status Solidi (B).

[81] Gharibshahian I., Orouji A.A., Sharbati S. (2021). Efficient Sb_2_(S,Se)_3_/Zn(O,S) solar cells with high open-circuit voltage by controlling sulfur content in the absorber-buffer layers. Sol. Energy.

[82] Barthwal S., Singh S., Chauhan A.K., Karuppannan R. (2023). Design and Simulation of CdS-Free Sb_2_(S, Se)_3_ Solar Cells with Efficiency Exceeding 20%. ACS Sustain. Chem. Eng..

