# Supplementary material for: An Optimization Path for Sb2(S,Se)3 Solar Cells to Achieve an Efficiency Exceeding 20%
Source: Nanomaterials (Basel). 2024 Sep 2;14(17):1433. doi: 10.3390/nano14171433 (PMC11397007; doi:10.3390/nano14171433)
Supplement: Supplementary file 1 [file nanomaterials-14-01433-s001.zip › nanomaterials-3165916-supplementary.pdf]

Supplementary material

An Optimization Path for $Sb_2(S,Se)_3$ Solar Cells to Achieve an Efficiency Exceeding 20%

Xiaoyong Xiong [1,2,3], Chao Ding [2,*], Bingfeng Jiang [3], Guanggen Zeng [1] and Bing Li [1,*]

1 College of Materials Science and Engineering, Sichuan University, Chengdu 610064, China;
2 Institute of New Energy and Low-Carbon Technology, Sichuan University, Chengdu 610065, China
3 College of Intelligent Systems Science and Engineering, Hubei Minzu University, Enshi 445000, China;
* Correspondence: dc1107@scu.edu.cn (C.D.); libing70@126.com (B.L.)

Table S1. Simulation parameters applied to different layers within the solar cell model.

Parameters (Unit)	FTO [1]	CdS	$Sb_2(S,Se)_3$	Spiro-OMeTAD
Thinckness (nm)	350	60	300	50
Bandgap E_g (eV)	3.6	2.44 [2]	1.43 [2]	3.1 [2]
Electron affinity χ (eV)	4.5	4.1 [3]	3.81 [2]	2.1 [2]
Relative dielectric permittivity ε_r	8.9	10[4, 5]	15 [6]	3 [7]
CB effective density of states N_C (cm^{-3})	2.2×10^{18}	2.2×10^{18}	2.2×10^{18}	2.2×10^{18}
VB effective density of states N_V (cm^{-3})	1.8×10^{19}	1.8×10^{19}	1.8×10^{19}	1.8×10^{19}
Electron mobility μ_n ($cm^2 \cdot V^{-1} \cdot s^{-1}$)	100	100 [4]	9.8 [8]	2×10^{-4} [7]
Hole mobility μ_p ($cm^2 \cdot V^{-1} \cdot s^{-1}$)	25	25 [4]	10 [8]	2×10^{-4} [7]
Donor density N_D (cm^{-3})	10^{20}	1.1×10^{18} [5,9]	0	0
Acceptor density N_A (cm^{-3})	0	0	3.14×10^{16} [10]	4×10^{18}[7]
Defect density N_t (cm^{-3})	10^{16}	10^{14}		10^{15}[11]

Table S2. Simulation parameters for bulk defects in $Sb_2(S,Se)_3$ [2].

Defect type	defect1	defect2
Eletron capture cross section σ_n (cm^2)	1.99×10^{-17}	5.91×10^{-17}
Hole capture cross section σ_p (cm^2)	1.99×10^{-17}	5.91×10^{-17}
Reference for defect energy level E_t	Above the highest E_v	Above the highest E_v
energy with respect to Reference (eV)	0.5	0.671
Defect density N_t (cm^{-3})	6.28×10^{12}	1.88×10^{13}

Table S3. Defect parameters at the interfaces of CdS/$Sb_2(S,Se)_3$ and $Sb_2(S,Se)_3$/spiro-OMeTAD .

Parameters (Unit)	CdS/$Sb_2(S,Se)_3$ interface	$Sb_2(S,Se)_3$/spiro-OMeTAD interface
Defect type	Neutral	Neutral
Eletron capture cross section (cm^2)	1×10^{-15}	1×10^{-19}
Hole capture cross section (cm^2)	1×10^{-15}	1×10^{-19}
Reference for defect energy level E_t	Above the highest E_v	Above the highest E_v
Energy with respect to Reference (eV)	0.6	0.6
Total trap density (cm^{-2})	2.5×10^{14}	1.4×10^{14}

Table S4. Performance Comparison of $Sb_2(S,Se)_3$ Solar Cells: This Work vs. Published Studies.

Device structure	Absorber thickness (μm)	V_{oc} (V)	J_{sc} (mA·cm^{-2})	FF (%)	PCE (%)	Ref.
FTO/CdS/$Sb_2(S,Se)_3$/Spiro-OMeTAD/Au	0.60	1.31	24.05	58.56	18.43	[12]
ITO/CdS/$Sb_2(S,Se)_3$/MoS_2/Mo	0.80	0.95	35.32	75.96	25.67	[13]
FTO/$Cd_{0.6}Zn_{0.4}S$/$Sb_2(S,Se)_3$/Spiro-OMeTAD/Au	0.50	0.88	26.67	74.22	17.43	[14]
FTO/ZnO/$Sb_2(S,Se)_3$/Cu_2O/Au	0.55	0.95	27.70	68.00	18.00	[15]
FTO/ZnO/$Sb_2(S,Se)_3$/NiO/Au	0.50	0.94	27.6	67.00	17.3	[15]
FTO/$Cd_{0.6}Zn_{0.4}S$/$Sb_2(S,Se)_3$/Cu_2O/Au	0.90	0.89	27.4	71.00	17.3	[15]
ITO/CdS/$Sb_2(S, Se)_3$/MnS/Au	0.30	0.72	25.29	69.54	12.70	[16]
Al:ZnO/$Cd_{0.6}Zn_{0.4}S$/TiO_2/$Sb_2(S,Se)_3$/$MoSe_2$/Mo	2.00	0.64	32.34	75.75	15.65	[17]
FTO/ZnSe/$Sb_2(S,Se)_3$/$CuSbS_2$/Au	0.50	0.93	28.64	74.54	20.01	[18]
FTO/CdS/$Sb_2(S,Se)_3$/Spiro-OMeTAD/Au	0.60	0.99	30.93	87.09	26.77	This work

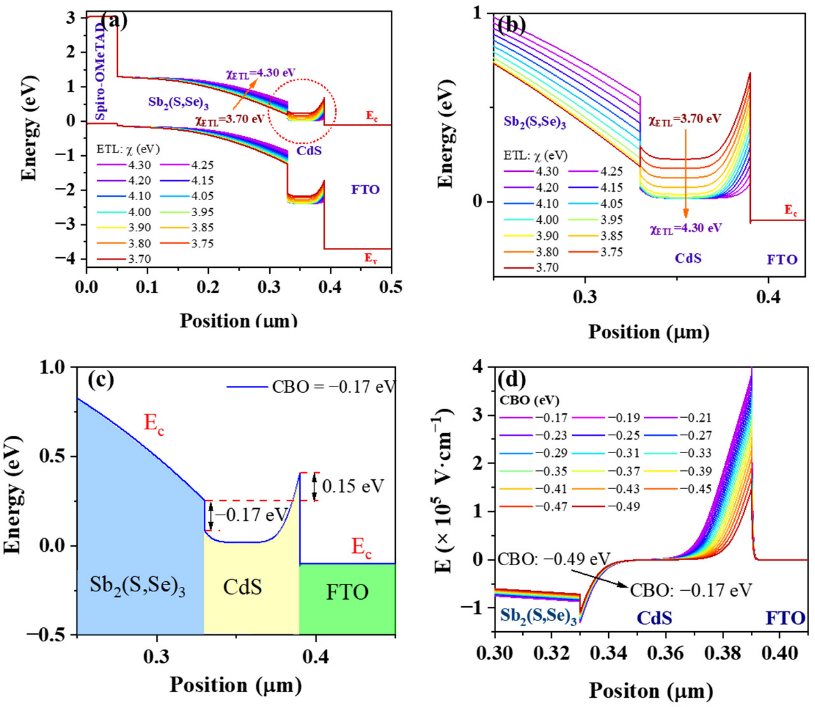

Figure S1. (a) Energy band diagram of $Sb_2(S,Se)_3$ solar cells under different χ_{ETL}. (b) The conduction band diagram of the dashed circle in (a). (c) The conduction band diagram at CBO = −0.17 eV. (d) The variation of built-in electric field at ETL/absorber and FTO/ETL interfaces with CBO varied from −0.49 eV to −0.17 eV.

References

[1] Zhou B.; Yin X.; Zhang J.; Zeng G.; Li B.; Zhang J.; Feng L. Numerical simulation of an innovative high efficiency solar cell with CdTe/Si composite absorption layer. *Optical Materials*, **2020**, 110. https://doi.org/10.1016/j.optmat.2020.110505.

[2] Chen X.; Che B.; Zhao Y.; Wang S.; Li H.; Gong J.; Chen G.; Chen T.; Xiao X.; Li J. Solvent-Assisted Hydrothermal Deposition Approach for Highly-Efficient $Sb_2(S,Se)_3$ Thin-Film Solar Cells. *Advanced Energy Materials*, **2023**, 13. https://doi.org/10.1002/aenm.202300391.

[3] Mao X.; Bian M.; Wang C.; Zhou R.; Wan L.; Zhang Z.; Zhu J.; Chen W.; Shi C.; Xu B. Ultrathin SnO_2 Buffer Layer Aids in Interface and Band Engineering for $Sb_2(S,Se)_3$ Solar Cells with over 8% Efficiency. *ACS Applied Energy Materials*, **2022**, 5, 3022-3033. https://doi.org/10.1021/acsaem.1c03660.

[4] Chen Y. J.; Wang Y. Y.; Wang R.; Hu X. B.; Tao J. H.; Weng G. E.; Zhao C. H.; Chen S. Q.; Zhu Z. Q.; Chu J. H.; et al. Importance of Interfacial Passivation in the High Efficiency of Sb2Se3 Thin-Film Solar Cells: Numerical Evidence. *Acs Applied Energy Materials*, **2020**, 3, 10415-10422. https://doi.org/10.1021/acsaem.0c01203.

[5] Zhou R.; Li X.; Wan L.; Niu H.; Wang H.; Yang X.; Wang X.; Hou J.; Xu J.; Xu B. Bulk Heterojunction Antimony Selenosulfide Thin-Film Solar Cells with Efficient Charge Extraction and Suppressed Recombination. *Advanced Functional Materials*, **2023**, 34, 2308021. https://doi.org/10.1002/adfm.202308021.

[6] Dong J.; Liu H.; Ding L.; Che B.; Xiao P.; Cao Z.; Liu Y.; Lou L.; Tang R.; Luo J.; et al. Lowest Open-Circuit Voltage Deficit Achievement to Attain High Efficient Antimony Selenosulfide Solar Cells. *Advanced Functional Materials*, **2023**, 34, 2309764. https://doi.org/10.1002/adfm.202309764.

[7] Khadir A. $Sb_2(S,Se)_3$-Based Thin Film Solar Cells: Numerical Investigation. *Acta Physica Polonica A*, **2023**, 144, 52-60. https://doi.org/10.12693/APhysPolA.144.52.

[8] Mamta; Maurya K. K.Singh V. N. Sb_2Se_3 versus Sb_2S_3 solar cell: A numerical simulation. *Solar Energy*, **2021**, 228, 540-549. https://doi.org/10.1016/j.solener.2021.09.080.

[9] Ngoupo A. T.; Ouédraogo S.; Zougmoré F.; Ndjaka J. M. B. Numerical analysis of ultrathin Sb_2Se_3-based solar cells by SCAPS-1D numerical simulator device. *Chinese Journal of Physics*, **2021**, 70, 1-13. https://doi.org/10.1016/j.cjph.2020.12.010.

[10] Xing Y.; Guo H.; Liu J.; Zhang S.; Qiu J.; Yuan N.; Ding J. High-efficiency $Sb_2(S,Se)_3$ solar cells with MoO_3 as a hole-transport layer. *Journal of Alloys and Compounds*, **2022**, 927. https://doi.org/10.1016/j.jallcom.2022.166842.

[11] Hajjiah A.; Gamal M.; Kandas I.; Gorji N. E.; Shehata N. DFT and AMPS-1D simulation analysis of all-perovskite solar cells based on $CsPbI_3/FAPbI_3$ bilayer structure. *Solar Energy Materials and Solar Cells*, **2022**, 248. https://doi.org/10.1016/j.solmat.2022.112026.

[12] Ayala-Mató F.; Vigil-Galán O.; Nicolás-Marín M. M.; Courel M. Study of loss mechanisms on $Sb_2(S_{1-x}Se_x)_3$ solar cell with n–i–p structure: Toward an efficiency promotion. *Applied Physics Letters*, **2021**, 118. https://doi.org/10.1063/5.0032867.

[13] Mamta; Kumar R.; Kumari R.; Maurya K. K.; Singh V. N. $Sb_2(S,Se)_3$-based photovoltaic cell with MoS_2 as a hole transport layer: a numerical investigation. *Materials Today Sustainability*, **2022**, 20, 100218. https://doi.org/10.1016/j.mtsust.2022.100218.

[14] Nicolás-Marín M. M.; Ayala-Mato F.; Vigil-Galán O.; Courel M. Simulation analysis of $Cd_{1-x}Zn_xS/Sb_2(Se_{1-x}S_x)_3$ solar cells with n-i-p structure. *Solar Energy*, **2021**, 224, 245-252. https://doi.org/10.1016/j.solener.2021.05.092.

[15] Nicolás-Marín M. M.; Vigil-Galán O.; Ayala-Mato F.; Courel M. Analysis of Hole Transport Layer and Electron Transport Layer Materials in the Efficiency Improvement of $Sb_2(Se_{1-x}S_x)_3$ Solar Cell. *physica status solidi (b)*, **2023**, 260, 2200342. https://doi.org/10.1002/pssb.202200342.

[16] Sekar K.; Mayarambakam S. Effect of Annealed and Non-Annealed Inorganic MnS Hole-Transport Layer for Efficient $Sb_2(S,Se)_3$ Solar Cells: A Theoretical Justification. *Physica Status Solidi B-basic Solid State Physics*, **2023**, 260, 2300087. https://doi.org/10.1002/pssb.202300087.

[17] Gharibshahian I.; Orouji A. A.; Sharbati S. Efficient $Sb_2(S,Se)_3/Zn(O,S)$ solar cells with high open-circuit voltage by controlling sulfur content in the absorber-buffer layers. *Solar Energy*, **2021**, 227, 606-615. https://doi.org/10.1016/j.solener.2021.09.039.

[18] Barthwal S.; Singh S.; Chauhan A. K.; Karuppannan R. Design and Simulation of CdS-Free $Sb_2(S,Se)_3$ Solar Cells with Efficiency Exceeding 20%. *ACS Sustainable Chemistry & Engineering*, **2024**, 12, 947-958. https://doi.org/10.1021/acssuschemeng.3c06210.
